# ﻿Unusual case of splenic sarcoidosis without morphological lesions detected by PET-CT in a patient with breast cancer: functional imaging between pitfalls and therapeutic guide

**DOI:** 10.3332/ecancer.2017.766

**Published:** 2017-09-08

**Authors:** Gaetano Paone, Simona Di Lascio, Andrea Azzola, Luca Mazzucchelli, Olivia Pagani

**Affiliations:** 1 Department of Nuclear Medicine and PET/CT Centre, Oncology Institute of Southern Switzerland, Bellinzona, Switzerland; 2 Department of Oncology, Oncology Institute of Southern Switzerland, Bellinzona, Switzerland; 3 Service of Pneumology, Department of Internal Medicine, EOC Switzerland; 4 Institute of Pathology, Locarno, Switzerland

**Keywords:** 18F-FDG, positron emission tomography, spleen sarcoidosis, breast cancer

## Abstract

A 60-year-old woman under treatment with letrozole for metastatic breast cancer underwent ^18^F-FDG PET-CT for restaging. A new widespread intense splenic FDG uptake without nodular lesions and multiple FDG-avid mediastinal and abdominal nodes were observed. Based on these findings, a nodal and transbronchial lung biopsy was performed. Histological results were compatible with sarcoidosis. The patient began steroid treatment and 6 weeks after a PET-CT showed normalisation of both splenic and nodal uptake. In our case, ^18^F-FDG PET-CT has been useful in detecting a rare case of splenic sarcoidosis without typical nodular lesions on CT images, impacting the patient’s treatment and prognosis.

## Introduction

Sarcoidosis is a benign systemic granulomatous disorder of unknown aetiology. The condition usually involves the lungs and mediastinal or hilar nodes [1]. One of the most frequent extra-thoracic manifestations is peripheral lymph node involvement, found in 8–15% of patients [2–4].

Blank *et al*. reported breast and cervical cancer and B-cell lymphoma as the most common malignancies in patients with sarcoidosis [1].

A retrospective report of 12 cases and a literature review showed that sarcoidosis frequently affects patients with breast cancer (32.3%) and must be considered in the differential diagnosis of the disease [5].

## Case report

**Figure 1.**
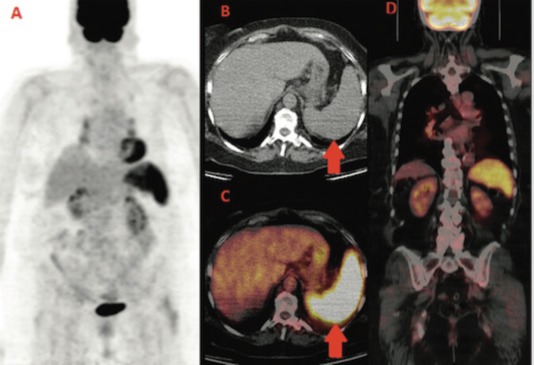
First F-FDG performed; intense and diffuse tracer uptake in the spleen and hilar lymph nodes.

We report the case of a 60-year-old woman diagnosed with left breast cancer in 2006. The histology showed an oestrogen (ER) and progesteron (PgR) receptor positive, HER2 negative, invasive lobular carcinoma, with some palpable left axillary nodes. The staging CT scan showed multiple mediastinal and abdominal lymph nodes. The patient was asymptomatic. Considering her breast cancer as metastatic, she received surgery on the primary tumour and axillary nodes but no radiation treatment and started palliative letrozole. The breast cancer’s stage was established as pT1c N3 M1. She was followed with clinical, biochemical evaluations and an annual CT scan that always showed stable disease. In the late 2013, for the first time, a ^18^F-FDG PET-CT was performed for disease status assessment. Images were acquired one hour after intravenous injection of 240 MBq (6.5 mCi) of ^18^F-FDG according to the body mass index. Maximum-intensity projection PET image (A), axial CT (B), and fused PET/CT images (C,D) (Figure 1) showed intense and diffuse tracer uptake in the spleen (red arrow), which had an homogeneous parenchymal structure without focal nodular lesions and, in mediastinal field, multiple FDG avid bilateral hilar lymph nodes characterised by greater lymph node enlargement on the right side.

**Figure 2.**
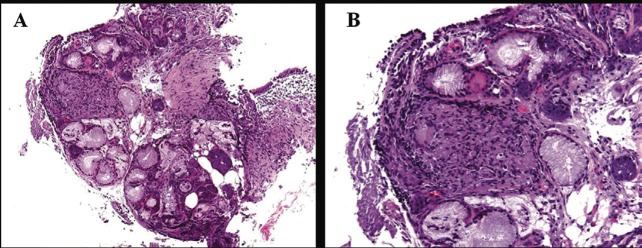
Histological features showing non-caseating granuloma between the mucinous bronchial glands (A) and submucosal granuloma consisting of a nodular cluster of epithelioid and giant cells (B).

Based on the ^18^F-FDG PET-CT findings, a transbronchial lung and lymph node biopsy were performed. Histological features were compatible with sarcoidosis showing noncaseating granuloma between the mucinous bronchial glands (A) and submucosal granuloma consisting of a nodular cluster of epithelioid and giant cells (B) (Figure 2).

**Figure 3.**
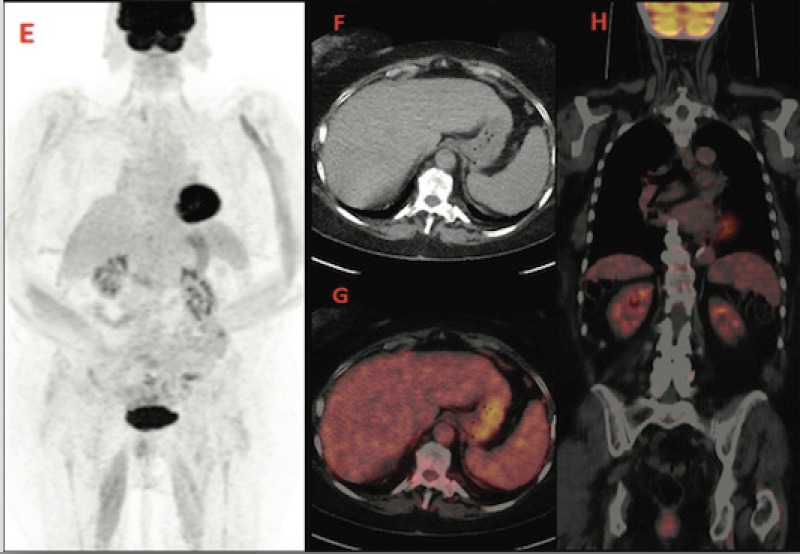
PET-CT after treatment that shows a rapid decrease of FDG uptake.

A control PET-CT performed after 6 weeks of prednisone showed a rapid decrease of FDG uptake in both nodes and spleen (images E,F,G,H) (Figure 3).

During all this time period, the patient had never been symptomatic for any respiratory or abdominal complaints.

Re-evaluating the clinical history of this patient, we concluded breast cancer was not metastatic at diagnosis and she could have received a curative multidisciplinary treatment upfront.

## Discussion

Examples of sarcoidosis or sarcoid-like reactions associated with malignancy have already been reported in the literature, in many cases as bilateral mediastinal lymph node involvement and sometimes as organ infiltration characterised by typical granulomas and/or nodular lesions [6–9]. The frequency of sarcoidosis in cancer patients is 4.4% higher than in the general population [10]. Cases of sarcoidosis mimicking metastatic breast cancer have already been described [11, 12]. There are several possible chronological associations between breast cancer and sarcoidosis. As previously reported, the two diseases may develop in tandem, or breast cancer may induce a sarcoidosis-like granulomatous response [13, 14].

The frequency of splenic involvement in sarcoidosis has been reported to be 10–50%, depending on whether it is detected on physical examination (5–14%), by a radiological test (33–53%), or a tissue biopsy (24–59%) [15].

A previous case report described a spleen sarcoidosis highlighted by ^18^F-FDG PET-CT and associated with 1,25-dihydroxyvitamin D [1,25(OH)2D]-mediated hypercalcemia [16]. In our case, ^18^F-FDG PET-CT has been crucial in detecting a spleen sarcoidosis without classical focal nodular lesions, therefore previously misdiagnosed as advanced breast cancer. The splenic and lymph node complete response after steroids has allowed a quick disease down staging. The patient was considered disease-free and PET-positive lymph nodes as false positive. This case underlines that the hybrid imaging can discover sarcoidosis and sarcoid-like reactions before and more specifically than conventional imaging [17]. The functional impairment highlighted by PET-CT may anticipate morphological alterations and therefore provide useful information for correct staging and early and appropriate therapeutic decisions. On the other hand, sarcoidosis is one of the known pitfalls in PET imaging, frequently causing false-positive results; expertise and knowledge are therefore key for a correct imaging interpretation.

## Conclusions

This clinical case emphasises the clinical utility of always performing biopsies of suspicious lesions, as reaffirmed by the third ESO-ESMO international consensus for advanced breast cancer [18] and shows that PET-CT can help in detecting unusual parenchymal sarcoidosis localisations.
